# Author’s reply to Mandarino et al.: The prophylactic use of endoscopic vacuum therapy for anastomotic dehiscence after rectal anterior resection—isit feasible for redo surgery?

**DOI:** 10.1007/s10151-022-02598-w

**Published:** 2022-02-26

**Authors:** N. C. Lehwald-Tywuschik, A. Alexander, N. Alkhanji, G. E. W. Flügen, S. Fung, A. Rehders, W. T. Knoefel

**Affiliations:** Departments of Surgery, University Hospital Duesseldorf and Heinrich-Heine-University Duesseldorf, Moorenstrasse 5, 40225 Düsseldorf, Germany

Dr Madarino et al. report on a novel application of prophylactic EVT treatment after redo surgery in case of anastomotic dehiscence [1]. The approach of combining redo surgery and enforcing the operative result though EVT is convincing and the result in this case is encouraging. Sufficient mobilization of the anastomotic bowel walls and preserving vascularisation of these margins will be crucial. We congratulate Dr Mandarino et al. on this innovative approach.
